# Circulating complement factor H levels are associated with disease severity and relapse in autoimmune hepatitis

**DOI:** 10.1016/j.jhepr.2022.100497

**Published:** 2022-04-29

**Authors:** Manabu Hayashi, Kazumichi Abe, Masashi Fujita, Atsushi Takahashi, Hideharu Sekine, Hiromasa Ohira

**Affiliations:** 1 Department of Gastroenterology, Fukushima Medical University, Fukushima, Japan; 2 Department of Immunology, Fukushima Medical University, Fukushima, Japan

**Keywords:** autoimmune hepatitis, relapse, complement system, factor H, MASP, AIH, autoimmune hepatitis, ALF, acute liver failure, ALT, alanine aminotransferase, AST, aspartate aminotransferase, C3, complement factor 3, C5, complement factor 5, IAIHG, International Autoimmune Hepatitis Group, MAC, membrane attack complex, MASP, mannose-binding lectin-associated serine protease, MBL, mannose-binding lectin, PRM, pattern recognition molecule, PT, prothrombin time, TB, total bilirubin

## Abstract

**Background & Aims:**

The complement system plays pivotal roles in innate immunity. Mannose-binding lectin-associated serine protease (MASP)-2 plays essential roles in the activation of the lectin complement pathway. Complement factor H acts as a critical negative regulator of the alternative complement pathway. The association of circulating MASP-2 and factor H with the clinical features of patients with autoimmune hepatitis (AIH) is unclear.

**Methods:**

A total of 63 patients with AIH were recruited for this study. The serum levels of MASP-2, factor H, and C3a were measured, and their associations with the clinical features of AIH were analyzed.

**Results:**

The circulating C3a levels were higher in patients with AIH than in the controls. The circulating MASP-2 and factor H levels were decreased depending on the severity of AIH. Multivariate logistic analysis showed that low circulating factor H levels were associated with features of severe AIH (odds ratio 0.36; 95% CI 0.15-0.84; *p =* 0.018). Multivariate Cox proportional hazards model analysis showed that low circulating factor H levels were associated with a high incidence of relapse (hazard ratio: 5.19; 95% CI 1.07–25.2; *p =* 0.041). Patients with low circulating factor H levels showed higher rates of relapse than the controls (log-rank, *p =* 0.006).

**Conclusion:**

Circulating factor H levels were associated with severe disease and with the incidence of relapse, suggesting a role for the complement system in the pathophysiology of AIH.

**Lay summary:**

Autoimmune hepatitis is an immune-mediated liver disease. Despite effective treatments, patients often relapse, which can lead to clinical deterioration and adverse outcomes. Herein, we studied the importance of the complement system (a form of innate immunity) in patients with autoimmune hepatitis. We found that the levels of a protein called factor H, which regulates the complement system, could be a potential biomarker of disease severity and relapse, and could even have therapeutic potential for patients with AIH.

## Highlights

∙ We measured serum MASP-2 and factor H in patients with AIH.
∙ Serum MASP-2 and factor H levels were lower in patients with severe AIH.
∙ Patients with AIH and low factor H before treatment showed a high rate of relapse.

## Graphical abstract

**Figure:**
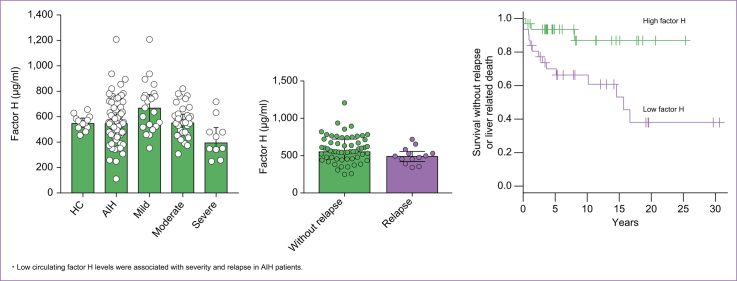

## Introduction

Autoimmune hepatitis (AIH) is an immune-mediated liver disease that leads to cirrhosis or liver failure.^1^ While remission can be achieved with immunosuppressive therapy, patients with AIH often relapse, and the incidence of relapse is associated with increased liver fibrosis and clinical deterioration.^2^ In total, 25-75% of patients with AIH present with acute disease, which can further progress to acute severe hepatitis or acute liver failure.^3^ A recent study on AIH revealed increased incidence and high mortality rates.^4^

The complement system plays an important role in maintaining biological homeostasis and in immune surveillance.^5^ The complement system can be activated through 3 pathways: the classical, lectin, and alternative pathways. These pathways involve cascade reactions of complement proteases to generate complement factor 3 (C3) convertase, which cleaves C3 into C3a and C3b. C3b generates complement factor 5 (C5) convertase, which cleaves C5 into C5a and C5b. C3a and C5a act as anaphylatoxins that accelerate the inflammatory response by binding to their receptors C3aR and C5aR, respectively.^6^^,^^7^ C3b and iC3b, proteolytically inactive C3b fragments, regulate humoral immunity by binding to complement receptors on B cells or follicular dendritic cells.^8^^,^^9^ Therefore, these complement split products generated by the activation of C3 and C5 modulate adaptive immunity.^10^^,^^11^ Furthermore, the C5b fragment can combine with C6, C7, C8, and C9 to form the C5b-9 membrane attack complex (MAC) on target surfaces, ultimately causing cell lysis.

Mannose-binding lectin-associated serine protease (MASP)-2 and MASP-3 are key enzymes for the activation of the lectin and alternative pathways, respectively.^12^^,^^13^ In the circulation, MASP-2, along with MASP-1, forms a complex with humoral pattern recognition molecules (PRMs), such as mannose-binding lectin (MBL), ficolins and collectins.^14^ Activation of the lectin pathway is initiated by the binding of PRM/MASP complexes to pathogen-associated molecular patterns or damage-associated molecular patterns. MASP-2 is then activated by autoactivated MASP-1 and cleaves C4 and C2 to generate the lectin pathway C3 convertase C4bC2a. Unlike MASP-1 and MASP-2, MASP-3 circulates in an active form. MASP-3 is thought to constantly cleave factor D of the alternative pathway and convert it to an active form in the circulation.^13^^,^^15^ Factor D cleaves factor B, which binds C3b to generate the alternative pathway C3 convertase C3bBb.

Factor H plays critical roles in the negative regulation of alternative pathway activation.^16^ Factor H inhibits C3bBb formation by competing with factor B for binding to C3b and accelerates irreversible C3bBb decay by displacing Bb.^17^ Factor H also acts as a cofactor for factor I-catalyzed C3b cleavage, degenerating iC3b, which cannot bind factor B. MASP-2 and factor H are mainly produced in the liver, and circulating complement factors are associated with several diseases, such as inflammatory, kidney, and infectious diseases, including coronavirus disease 2019.[18], [19], [20], [21]

Previous studies suggested a critical role of the complement system in liver disease. The expression of complement receptors on Kupffer cells is needed for efficient phagocytosis.^22^ Both genetic C5 deficiency and a C5a receptor antagonist were shown to ameliorate liver fibrosis in CCl_4_-treated mice.^23^ An anti-C5 antibody was effective at treating concanavalin A-induced acute liver failure (ALF) in mice.^24^ In patients with fulminant hepatitis and acute hepatitis, hepatocytes surrounding necrotic areas exhibited positive MAC staining.^25^ Genetic factor H-deficient mice spontaneously developed liver injury and hepatic tumors.^26^ Moreover, deposition of complement activation products, such as C4d, C3d, and C5b-9 (MAC), was observed on the hepatocytes of patients with AIH,^27^ and complement-mediated hepatocyte injury was observed in a murine model of autoantibody-induced hepatitis.^28^ While these results suggest that complement activation is involved in the development of AIH, whether complement regulators contribute to the pathophysiology of AIH remains unclear.

Circulating complement factor levels are associated with disease activity, such as systemic lupus erythematosus and IgA nephropathy.^18^^,^^29^ Serum MASP-2 levels in patients with chronic hepatitis C (CHC) were associated with the severity of fibrosis and response to interferon treatment.^30^ High C5a or C5b-9 levels in the pretreatment serum of patients with CHC were associated with a high rate of HCV RNA clearance by direct-acting antivirals.^31^ In this study, we measured the serum levels of factor H, a major plasma regulator acting in the alternative pathway, in patients with AIH and analyzed their association with clinical features. We also analyzed the associations of the serum levels of C3a or MASP-2 with the clinical features of AIH.

## Patients and methods

### Patients

In this study, patients with AIH diagnosed at Fukushima Medical University Hospital (Fukushima, Japan) between September 1988 and June 2017 were included. The diagnosis of AIH was defined according to the revised and simplified International Autoimmune Hepatitis Group (IAIHG) scoring system.^32^^,^^33^ Patients with hepatitis B or hepatitis C and those who consumed significant amounts of alcohol (20 g/day in women and 60 g/day in men) were excluded. We excluded patients with suspected alcoholic liver injury even if the patient did not exceed the limit of alcohol intake. Patients with acute liver failure were excluded from this study. In total, 63 patients with AIH were included in this study. We also measured serum levels of complement proteins in patients with CHC (n = 20), drug-induced liver injury (DILI) (n = 20), liver cirrhosis (n = 40), and healthy controls (HCs) (n = 17).

Disease severity was classified according to the diagnosis and treatment guide for AIH in Japan.^34^ Briefly, severe cases were defined as those that met at least one of the following criteria: (1) clinical signs (hepatic encephalopathy or a reduction in or disappearance of hepatic dullness); (2) clinical laboratory test results (aspartate aminotransferase [AST] or alanine aminotransferase [ALT] >200 U/L, total bilirubin [TB] >5 mg/dl, or prothrombin time [PT] <60%); and (3) imaging test findings (hepatic atrophy or a heterogeneous liver parenchyma pattern). Acute AIH was defined by the presence of acute-onset symptoms (e.g., jaundice, fatigue and anorexia) in conjunction with a bilirubin level >5 mg/dl and/or a serum ALT level greater than 10-fold higher than the upper limit of normal. AIH relapse was defined as an increase in the transaminase level to more than twice the upper limit of normal (ALT level >90 U/L) after the induction of biochemical remission.^32^ Biochemical remission was defined as the normalization of aminotransferases and IgG.^35^

All patients agreed to serum and histological testing, and written informed consent was obtained. The study protocol conformed to the ethical guidelines of the 1975 Declaration of Helsinki and was approved for the use of opt-out consent by the ethics committee of Fukushima Medical University School of Medicine.

### Measurement of serum complement protein levels

All samples from AIH patients were collected at the time of diagnosis before the initiation of immunosuppressive therapy. Serum samples were stored at -20°C. The serum levels of C3a, MASP-2, and factor H were measured using commercial enzyme-linked immunosorbent assay (ELISA) kits according to the manufacturer’s protocol. All ELISA kits were purchased from Hycult Biotech (Uden, the Netherlands).

### Histological analysis

Among 63 patients, 60 underwent percutaneous liver biopsy using ultrasound guidance with an 18-gauge needle. Of the remaining three patients, two were diagnosed with liver cirrhosis, and one had poor health conditions and did not undergo a liver biopsy. The histological findings related to hepatitis were analyzed in 59 of the 60 patients because two patients lacked sufficient data. The liver histological findings were assessed by pathologists who were blinded to the clinical data. The hepatitis and fibrosis stages were graded according to the classification method provided by Scheuer and Desmet *et al.*,^36^^,^^37^ with scores ranging from 0 to 4. The levels of complement regulators were compared between patients with mild (hepatitis or fibrosis grade from 0 to 2) and severe (hepatitis or fibrosis grade of 3 or 4) disease as determined by histological findings.

### Statistical analysis

Quantitative variables are presented as the median and interquartile range. Differences in quantitative variables between the groups were compared by the Mann–Whitney *U* test. Correlations between serum complement protein levels and laboratory data were assessed by Spearman’s rank correlation test. Associations between serum levels of complement factors and storage periods were analyzed using Student’s t test and Pearson’s correlation test. Receiver operating characteristic (ROC) curves were built to assess the ability of serum MASP-2 levels to distinguish AIH from DILI. The AUROC was used to determine predictive power. Univariate and multivariate logistic regression analyses of the factors related to AIH severity were performed. Complement factors, ALT, and TB were included in a multivariate logistic regression analysis. Univariate and multivariate Cox proportional hazards models were used to assess associations with the incidence of relapse. Complement factor, TB, PT and prednisolone (PSL) withdrawal were included in a multivariate Cox proportional hazard model. Survival curves of patients without relapse were constructed using the Kaplan–Meier method, and the incidence of relapse in patients with AIH was analyzed using the log–rank test. Differences were considered statistically significant at *p* <0.05. Data analyses were carried out using Prism 7.0 software (GraphPad Software, La Jolla, CA, USA) and EZR (Saitama Medical Center, Jichi Medical University, Saitama, Japan),^38^ a graphical user interface for R software (The R Foundation for Statistical Computing, Vienna, Austria).

## Results

### Patient characteristics

The characteristics of the patients included in this study are shown in Table 1. The median age was 57 years, and 89% of the patients were female. The median follow-up period was 5.3 years (interquartile range: 3.0–13.6 years). Four patients were negative for anti-nuclear antibodies (6.3%), and 1 patient was positive for anti-liver kidney microsomal antibodies. The median IAIHG score was 17, and the median simplified score was 7. Fifty-eight patients were treated with PSL therapy, and 16 patients were treated with azathioprine in combination with PSL. Among 63 patients, 10 patients were classified as having severe AIH (15.8%). There were 34 (53%) patients with AIH in the acute phase. Fifty-nine patients achieved biochemical remission, but 15 patients experienced a relapse. We analyzed the association between relapse and azathioprine therapy using Fisher's exact test. The rates of azathioprine therapy in patients with and without relapse were 20% (3/15) and 27% (13/48), respectively (*p =* 0.74). During the follow-up period, 2 patients died due to liver failure. The characteristics of the patients with and without relapse are also shown in Table 1.

**Table 1:** **Patient characteristics**.

Variables	All patients	Without relapse	With relapse
Number of patients	63	48	15
Age at diagnosis	57 (47-65)	59 (52-66)	49 (35-57)
Sex, male/female	7/56	6/42	1/14
Laboratory data			
Aspartate aminotransferase, U/L	319 (73-684)	222 (78-559)	482 (67-1041)
Alanine aminotransferase, U/L	229 (81-786)	228 (108-659)	343 (58-771)
Alkaline phosphatase, U/L	407 (312-575)	406 (296-657)	408 (359-427)
Total bilirubin, mg/dl	2.0 (1.0-8.1)	1.7 (1.0-7.8)	2.6 (1.0-9.4)
Platelet count, ×10^4^/μl	15.8 (13.8-21.4)	16.3 (13.8-22.1)	14.2 (13.7-18.7)
Prothrombin time, %	75 (58-91)	77 (62-91)	69 (50-90)
IgG, mg/dl	2,490 (1,975-3,037)	2,502 (2,072-3,081)	2,326 (1,800-2,816)
Albumin, g/dl	3.5 (2.9-3.9)	3.5 (3.1-4.0)	3.3 (2.7-3.7)
ANA (≤×40/×80/≥×160)	4/9/50	3/5/40	0/4/11
Severity (mild/moderate/severe)	21/32/10	17/24/7	4/8/3
Histological findings			
Histological fibrosis (0/1/2/3/4) (n = 60)	7/7/21/15/10	5/6/17/11/8	2/1/4/4/2
Grade of interface hepatitis (0/1/2/3/4) (n = 59)	0/7/29/21/1	0/5/23/16/1	0/2/6/5/0
Scoring			
IAIHG score	17 (14-22)	17 (14-19)	16 (14-17)
Simplified score	7 (7-7)	7 (7-7)	7 (6-7)

Quantitative variables are presented as the median and interquartile range.

ANA, anti-nuclear antibody; IAIHG, International Autoimmune Hepatitis Group.

### Serum levels of C3a, MASP-2, and factor H in patients with AIH

**Fig. 1:**
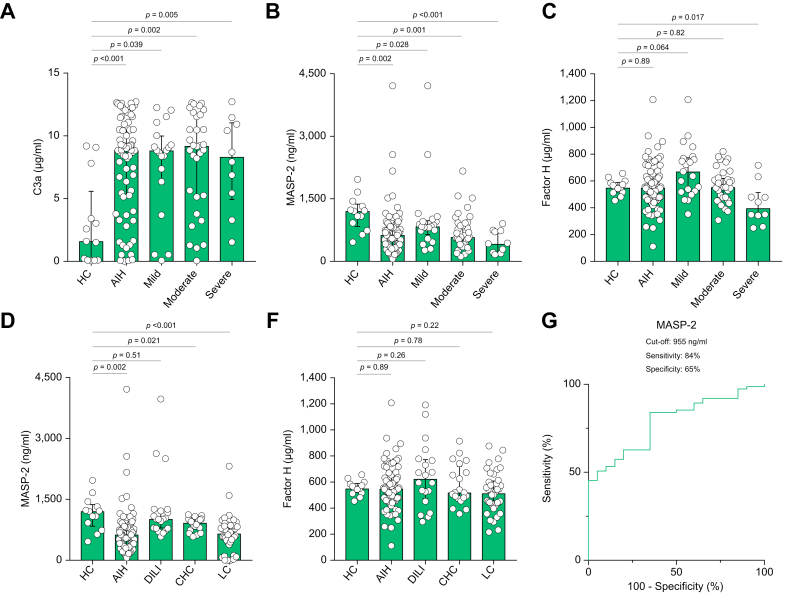
Serum C3a, MASP-2, and factor H levels in AIH patients. The figures show the serum levels of C3a (A), MASP-2 (B), and factor H (C) in AIH patients and HCs. The serum levels of complement proteins are also shown depending on the severity of AIH. The severity of AIH was classified as mild, moderate, or severe. The serum levels of MASP-2 (D) and factor H (E) in patients with AIH, CHC, DILI, and liver cirrhosis are shown (Mann–Whitney U test). (F) ROC curves for distinguishing AIH from DILI by MASP-2 levels. AIH, autoimmune hepatitis; CHC, chronic hepatitis C; DILI, drug-induced liver injury; HC, healthy control; MASP, mannose-binding lectin-associated serine protease; ROC, receiver operating characteristic.

We analyzed the association between serum levels of complement factors and storage periods. Serum levels of C3a, Factor H, and MASP-2 did not show a significant correlation with storage periods (C3a; r = -0.027, *p =* 0.82. Factor H; r = -0.22, *p* = 0.079. MASP-2; r = -0.19, *p =* 0.12). Next, we compared serum levels of complement factors in long storage periods and those of short storage periods. The cut-off value was 145 months, which is the median storage period in this study. Serum levels of C3a, Factor H, and MASP-2 did not show significant differences between long and short storage periods (serum C3a in short storage periods *vs.* serum C3a in long storage periods; 8.16 ± 3.85 μg/ml *vs.* 7.93 ± 3.82 μg/ml, *p =* 0.81. Serum Factor H in short storage periods *vs.* serum Factor H in long storage periods; 592 ± 186 μg/ml *vs.* 542 ± 144 μg/ml, *p =* 0.248. Serum MASP-2 in short storage periods *vs.* serum MASP-2 in long storage periods; 820 ± 714 μg/ml *vs.* 694 ± 539 μg/ml, *p =* 0.43). There was no significant association between these serum complement levels and storage periods. Circulating C3a is generated by the cleavage of C3 via complement pathway activation. First, we measured the serum C3a levels in patients with AIH and in HCs. The serum C3a levels in the AIH group were significantly higher than those in the HC group (8.86 [5.25-10.5] μg/ml *vs.* 1.60 [0.09-5.61] μg/ml, *p <*0.001) (Fig. 1A). Next, we measured the serum MASP-2 and factor H levels in the AIH and HC groups. The serum MASP-2 levels in the AIH group were significantly lower than those in the HC group (631 [353-888] ng/ml *vs.* 1,204 [831-1,383] ng/ml, *p <*0.001) (Fig. 1B). The serum factor H levels were not significantly different between the AIH and HC groups (547 [455-670] μg/ml *vs.* 549 [515-591] μg/ml, *p =* 0.89) (Fig. 1C).

We further analyzed the associations of serum C3a, MASP-2, and factor H levels with the severity of AIH. The serum C3a levels in the AIH group were significantly higher than those in the control group at all AIH severities (HC, 1.60 μg/ml; mild AIH, 8.83 μg/ml; moderate AIH, 9.19 μg/ml; severe AIH, 8.32 μg/ml) (Fig. 1A). Although the difference was not statistically significant, the serum C3a levels in the severe AIH group tended to be lower than those in the mild and moderate AIH groups. On the other hand, the serum MASP-2 levels in the AIH group were significantly lower than those in the HC group depending on the severity of AIH (HC, 1,204 ng/ml; mild AIH, 837 ng/ml; moderate AIH, 583 ng/ml; severe AIH, 422 ng/ml) (Fig. 1B). Interestingly, the serum factor H levels in the AIH group were significantly different from those in the HC group depending on the severity of AIH (HC, 549 μg/ml; mild AIH, 670 μg/ml; moderate AIH, 554 μg/ml; severe AIH, 396 μg/ml) (Fig. 1C). Serum factor H levels in the mild AIH group were higher than those in the HC group (*p =* 0.064). Conversely, the serum factor H levels in the severe AIH group were significantly lower than those in the HC group (*p =* 0.017). On the other hand, the serum factor H levels were not significantly different between the moderate AIH and HC groups (*p =* 0.89). These results suggest that serum levels of complement protein vary significantly depending on the severity of AIH.

We measured serum MASP-2 and factor H levels in patients with liver diseases other than AIH, such as CHC, DILI, and cirrhosis without AIH (Fig. 1D,E). Serum levels of MASP-2 in patients with CHC (921 [758-1,012] ng/ml, *p =* 0.021) or cirrhosis (656 [445-867] ng/ml, *p <*0.001) were lower than those in HCs. Serum levels of MASP-2 in patients with DILI did not show significant differences compared with those of HCs (MASP-2: 1,016 [785-1,246] ng/ml, *p =* 0.51). Serum levels of factor H in patients with CHC (519 [475-725] μg/ml, *p* = 0.78), DILI (624 [470-775] μg/ml, *p =* 0.26), or cirrhosis (512 [423-610] μg/ml, *p =* 0.22) did not show significant differences compared with those of HCs. Serum levels of MASP-2 in patients with AIH were significantly lower than those in patients with DILI (631 ng/ml *vs.* 1,016 ng/ml, *p <*0.0001). The AUROC of the serum MASP-2 levels for distinguishing AIH and DILI was 0.784. The sensitivity and specificity of serum MASP-2 levels for recognizing AIH or DILI according to the cut-off value of 955 ng/ml were 84% and 65%, respectively.

### Associations of the serum MASP-2 and factor H levels with laboratory test values and hepatic histological findings

**Fig. 2:**
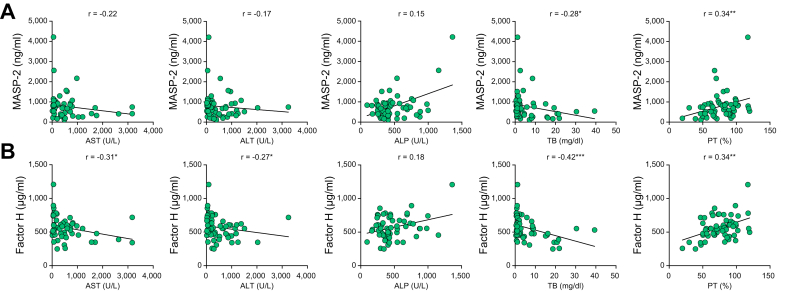
Correlations between serum complement protein levels and laboratory test values. The correlations of the serum MASP-2 (A) and factor H (B) levels with the AST, ALT, ALP, TB and PT test values are shown. Asterisks represent significant differences at ∗*p* < 0.05, ∗∗*p* < 0.01, or ∗∗∗*p* < 0.001 (Spearman’s rank correlation test). AST, aspartate aminotransferase; ALT, alanine aminotransferase; ALP, alkaline phosphatase; TB, total bilirubin; PT, prothrombin time; MASP, mannose-binding lectin-associated serine protease.

Next, we analyzed the associations of the serum MASP-2 and factor H levels with the AST, ALT, ALP, TB and PT test values in patients with AIH. The serum MASP-2 levels were significantly correlated with TB (r = -0.289, *p =* 0.026) and PT (r = 0.345, *p =* 0.008) (Fig. 2A). On the other hand, the serum factor H levels were significantly correlated with TB (r = -0.42, *p <*0.001) and PT (r = 0.34, *p =* 0.007) as well as with AST (r= -0.31, *p =* 0.014) and ALT (r = -0.27, *p =* 0.032) (Fig. 2B). In addition, the serum levels of these complement proteins were not significantly correlated with the serum IgG levels (data not shown). These results indicated that serum levels of MASP-2 and factor H correlated with laboratory test values associated with liver injury.

**Fig. 3:**
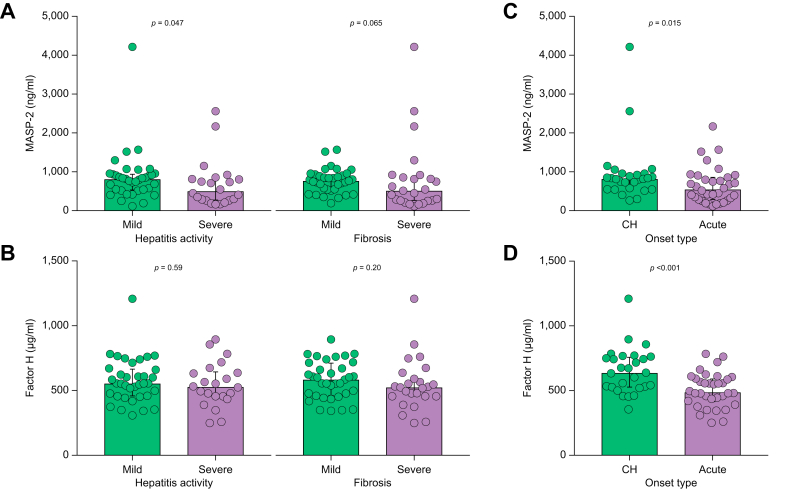
Serum complement protein levels in AIH patients as determined by histological classification and type of onset. The serum MASP-2 (A and C) and factor H (B and D) levels are shown (Mann–Whitney U test). The serum complement protein levels were compared between subjects with different histological classifications (A and B, hepatitis activity and fibrosis) and types of onset (C and D chronic and acute hepatitis phases). CH, chronic hepatitis phase; MASP, mannose-binding lectin-associated serine protease.

We further analyzed the associations of serum MASP-2 and factor H levels with hepatic histological findings in patients with AIH. The serum MASP-2 levels in patients with severe histological hepatitis were significantly lower than those in patients with mild histological hepatitis (Fig. 3A). The serum factor H levels were not significantly correlated with the hepatic histological findings (Fig. 3B). We also analyzed the associations of the serum MASP-2 and factor H levels with the onset type of AIH (Fig. 3C,D). The serum MASP-2 and factor H levels in patients with AIH in the acute phase were significantly lower than those in AIH patients in the chronic phase (MASP-2: 548 [299-841] ng/ml *vs.* 817 [552-954] ng/ml, *p =* 0.015. Factor H: 486 [422-589] μg/ml *vs.* 637 [527-748] μg/ml, *p* <0.001) (Fig. 3C,D).

### Associations of serum MASP-2 and factor H levels with the severity of AIH

As shown in Fig. 1, the serum complement protein levels varied depending on the severity of the disease in patients with AIH. To determine the complement proteins associated with the severity of AIH, we performed univariate and multivariate logistic analyses of the serum levels of MASP-2 and factor H and disease severity in patients with AIH. Multivariate logistic regression analyses, including ALT and TB, were performed. As shown in Table 2, low serum factor H levels were significantly associated with severe AIH (odds ratio 0.36; 95% CI 0.15-0.84; *p =* 0.018). On the other hand, serum MASP-2 levels were not significantly associated with severe AIH.

**Table 2:** **Univariate and multivariate logistic analyses of complement proteins influencing the severity of autoimmune hepatitis**.

	Univariate			Multivariate∗		
	Odds ratio	95% CI	*p* value	Odds ratio	95% CI	*p* value
MASP-2, 100 ng/ml	0.78	0.60-1.00	0.053	0.78	0.57-1.06	0.11
Factor H, 100 μg/ml	0.40	0.21-0.77	0.006	0.36	0.15-0.84	0.018

MASP, mannose-binding lectin-associated serine protease.

∗ Adjusted alanine aminotransferase and total bilirubin.

### Associations of serum MASP-2 and factor H levels with AIH relapse

**Fig. 4:**
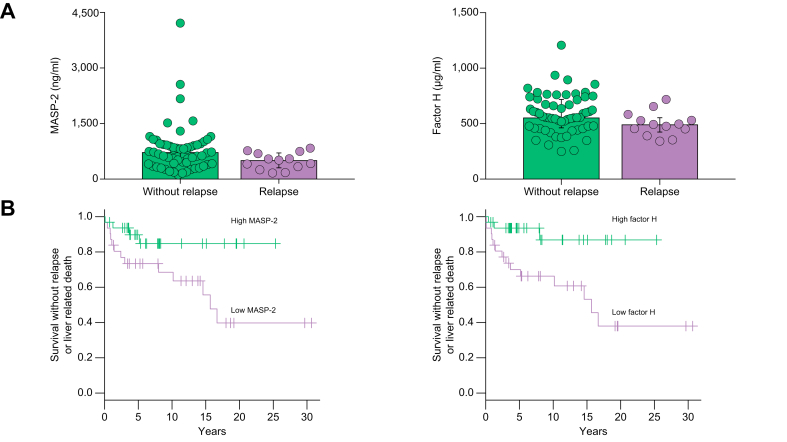
Associations of serum MASP-2 and factor H levels with AIH relapse. The figures show the differences in the complement protein levels between patients with and without relapse (Mann–Whitney U test) (A). Survival curves of subjects without relapse were constructed using Kaplan–Meier plots (log–rank test) (B). MASP, mannose-binding lectin-associated serine protease.

Finally, we analyzed the associations of serum MASP-2 and factor H levels with AIH relapse. The serum MASP-2 and factor H levels in patients with AIH who relapsed were significantly lower than those in patients who did not relapse (MASP-2: 514 [295-718] ng/ml *vs.* 730 [408-934] ng/ml, *p =* 0.032. Factor H: 494 [421-556] μg/ml *vs.* 555 [458-721] μg/ml, *p =* 0.044) (Fig. 4A). The Kaplan–Meier plot showed a higher rate of relapse in the patients with low serum levels of factor H (log-rank *p =* 0.006) or MASP-2 (log-rank *p =* 0.030) than in the other patients (Fig. 4B). In univariate Cox hazard analysis, low serum factor H levels and low serum MASP-2 levels were significantly associated with the relapse of AIH (Table 3). Furthermore, low serum factor H levels were significantly associated with AIH relapse features as determined by multivariate Cox hazard analysis (hazard ratio 5.19; 95% CI 1.07-25.2; *p =* 0.041).

**Table 3:** **Univariate and multivariate Cox proportional analyses of complement proteins influencing autoimmune hepatitis relapse**.

	Univariate			Multivariate∗		
	Hazard ratio	95% CI	*p* value	Hazard ratio	95% CI	*p* value
MASP-2, ≤709 ng/ml	3.24	1.05-9.98	0.040	3.54	0.92-13.6	0.065
Factor H, ≤553 μg/ml	4.74	1.36-16.55	0.014	5.19	1.07-25.2	0.041

MASP, mannose-binding lectin-associated serine protease.

∗ Adjusted total bilirubin, prothrombin time, and prednisolone withdrawal.

## Discussion

In this study, we observed that patients with AIH had significantly higher circulating levels of C3a than HCs, suggesting that the complement system is activated. Paradoxically, patients with severe AIH tended to have lower circulating levels of C3a than those with mild or moderate AIH, suggesting the consumption of serum C3 in these patients, as reported previously.^6^ We also observed significantly lower circulating levels of MASP-2 in patients with AIH than in HCs and patients with DILI. Interestingly, the circulating levels of factor H, a negative regulator of the complement alternative pathway, were significantly correlated with several laboratory parameters, such as AST, ALT, TB, and PT. Furthermore, circulating factor H levels were associated with disease severity and with the incidence of relapse in patients with AIH.

Further elucidation of the mechanism underlying the complement system in recent years has revealed that dysregulation or overactivation of the complement system is associated with several inflammatory diseases. It is known that factor H-deficient (*fH*^*-/-*^) mice spontaneously develop retinal abnormalities and nephritis due to overactivation of the complement system.^39^^,^^40^ Recent reports suggest an important protective role of factor H in chronic liver injury, and *fH*^*-/-*^ mice spontaneously develop liver injury due to excessive activation of the complement alternative pathway.^26^ Abundant infiltration of immune cells, especially monocytes and CD8-positive T cells, was observed in the livers of *fH*^*-/-*^ mice. Monocytes and CD8-positive T cells play important roles in the pathophysiology of AIH. The serum levels of soluble CD163, which is a biomarker of activated macrophages, are elevated in patients with AIH compared with HCs, and sustained elevation of soluble CD163 is associated with an incomplete response to treatment.^41^ Emperipolesis in AIH is predominantly mediated by CD8-positive T cells, and emperipolesis of CD8-positive T cells induces the expression of cleaved caspase-3 and apoptosis.^42^ Our results suggest that inappropriate complement activation due to insufficient circulating levels of factor H influences the pathophysiology of AIH via the modulation of immune cell responses.

In our study, circulating factor H levels were decreased depending on the severity of AIH, and patients with severe AIH had significantly lower circulating factor H levels than HCs. Interestingly, patients with mild AIH had significantly higher circulating factor H levels than HCs. These results suggest that the production of factor H is upregulated to suppress inappropriate complement activation in patients with mild AIH. In a previous study on patients with AIH, immunohistochemistry analysis revealed C3d deposition on hepatocytes but not MAC deposition.^27^ This finding may be associated with the role of circulating factor H because factor H is a cofactor for the factor I-catalyzed cleavage of C3b to iC3b, which is further cleaved to C3d. Factor H is also an important inactivator of the alternative pathway component C3 convertase C3bBb, which generates C5 convertase (C3bBbC3b) and is ultimately involved in the formation of C5b-9 (MAC) on the cell surface. Therefore, the upregulation of factor H may efficiently inhibit the formation of MACs on hepatocytes in patients with mild AIH. Our results also demonstrate that circulating factor H levels are associated with the severity of liver dysfunction independent of ALT and TB. Furthermore, circulating factor H levels were negatively correlated with serum aminotransferase levels. Complement activation features, such as increased circulating C5a levels and MAC formation on hepatocytes, were detected in animal models and patients with acute liver failure.^25^^,^^43^ In our study, the circulating factor H levels were lower in patients with AIH in the acute phase than in patients in the chronic phase. These findings suggest that low circulating levels of factor H in patients with AIH are associated with disease progression and with the development of severe AIH.

While corticosteroid therapy is effective and improves liver injury in patients with AIH, relapse often occurs during the follow-up period. Although relapse of AIH is usually asymptomatic, it is associated with disease progression and the deterioration of liver function.^2^ PSL withdrawal, high TB levels, and low PT levels have been reported as risk factors for relapse in patients with AIH.^44^^,^^45^ In our study, low circulating levels of factor H were associated with relapse independent of PSL withdrawal, TB, and PT. Several other risk factors for relapse in patients with AIH have been reported, such as a shorter duration of inactive disease prior to treatment withdrawal, increased serum ALT and IgG levels at drug withdrawal, and delayed biochemical remission.[46], [47], [48] These findings suggest that the incidence of relapse is associated with sustained inflammatory and immune responses. Low circulating levels of factor H in patients with AIH may be associated with relapse due to the insufficient suppression of inflammatory and immune responses after treatment. MASP-1/3-deficient mice were shown to lack lectin and alternative pathway activation, but mice with the combined deficiencies of factor H, MASP-1 and MASP-3 showed uncontrolled complement activation and developed complement-mediated renal disease.^49^ These results indicate that the presence of factor H is more important than the absence of MASP-1 and/or MASP-3 in the prevention of complement-mediated renal disease. In other words, factor H plays important protective roles in the development of complement-mediated renal disease. Therefore, the administration of factor H may be a therapeutic option for patients with uncontrolled AIH associated with low circulating levels of factor H. There were no significant differences between the rate of relapse in patients with AIH treated with prednisolone monotherapy and that of patients treated with prednisolone and azathioprine therapy in this study. This result may be associated with the reason for initiating azathioprine therapy because we initiated azathioprine therapy for two reasons: one reason was to induce remission, and the other was to treat relapse.

In this study, serum levels of complement factors did not correlate with IgG levels. High IgG levels are a hallmark of AIH, and elevation of IgG levels indicates ongoing inflammatory activity in patients with AIH. On the other hand, AIH with acute presentation shows lower IgG levels than chronic AIH. More than half of the patients had IgG levels below the upper limit of normal.^50^ In patients with AIH with acute presentation, the levels of ALT or PT and the rate of relapse did not show a significant association with the levels of IgG in a previous study. In this study, 34 patients (53%) were diagnosed with AIH with acute presentation. The lack of correlation between serum complement factors and IgG levels in this study may be associated with the rate of AIH with acute presentation.

Complement activation is initiated via 3 complement activation pathways: the classical, lectin, and alternative pathways. The classical complement pathway is initiated by antigen-antibody complexes with the antibody isotypes IgG and IgM.^5^ However, the other 2 pathways can be activated without the involvement of IgG. MASP-2 plays an essential role in activation of the lectin pathway, and factor H regulates activation of the alternative pathway. Interestingly, serum MASP-2 and factor H levels in AIH with acute presentation showed significant differences compared to those in chronic AIH. Serum levels of MASP-2 and factor H may be associated with the liver injury pathway independent of IgG.

MASP-2 is a serine protease that facilitates activation of the lectin pathway by cleaving C2 and C4.^12^ Because MASP-2 is mainly produced in the liver, decreased circulating MASP-2 levels in patients with AIH may be associated with decreased hepatic protein synthesis due to liver dysfunction. Low serum levels of MASP-2 in patients with cirrhosis in this study may be associated with decreased hepatic protein synthesis due to liver dysfunction. Another possible mechanism by which circulating MASP-2 levels could be decreased in patients with AIH is via the consumption of MASP-2 through lectin pathway activation. In a previous report, the peripheral levels of MASP-2 in patients with myocardial infarction were significantly lower than those in HCs, and the coronary levels of MASP-2 were negatively correlated with the myocardial necrosis marker.^51^ In our study, the circulating MASP-2 levels were significantly lower in patients with mild AIH than in HCs. Furthermore, serum MASP-2 levels in patients with AIH were significantly lower than those in patients with DILI. These results suggest that low circulating MASP-2 levels in patients with AIH may be associated with the consumption of MASP-2 via lectin pathway activation.

There are several limitations in this study. The major limitation of this study is the small sample size, although we did demonstrate an association between factor H and the clinical features of patients with AIH. The inclusion period in this study was very long. Although serum C3a, MASP-2, and factor H levels did not show a significant association with storage periods, it has been reported that the serum levels of complement factors may change during storage.^52^ We did not histologically analyze complement deposition in the liver. In addition, the complement system plays both harmful and protective roles in several conditions. A recent report showed the protective effect of MBL against liver injury induced by carbon tetrachloride in mice.^53^ More analyses are needed to elucidate the roles of factor H in patients with AIH.

In summary, circulating factor H levels were associated with disease severity and relapse of AIH. Our results suggest that factor H plays important roles in AIH, but this has not been fully clarified. Further study is needed to elucidate the contribution of the complement system to the pathophysiology of AIH.

## Financial support

No funding was received for this study.

## Authors’ contributions

M.H., H.S. and H.O. designed the study. M.H. performed the research, wrote the manuscript and prepared figures. M.H., K.A., M.F., and A.T. acquired and analyzed the data. All authors reviewed the manuscript.

## Data availability statement

All data that support the findings in this study can be found within this article.

## Conflict of interest

None of the authors have any conflicts of interest to declare.

Please refer to the accompanying ICMJE disclosure forms for further details.
