# Supplementary material for: Circulating complement factor H levels are associated with disease severity and relapse in autoimmune hepatitis
Source: JHEP Rep. 2022 Apr 29;4(7):100497. doi: 10.1016/j.jhepr.2022.100497 (PMC9167978; doi:10.1016/j.jhepr.2022.100497)
Supplement: Multimedia component 1 [file mmc1.docx]

**JHEP Reports**

**CTAT methods**

Tables for a “Complete, Transparent, Accurate and Timely account” (CTAT) are now mandatory for all revised submissions. The aim is to enhance the reproducibility of methods.

- Only include the parts relevant to your study
- Refer to the CTAT in the main text as ‘Supplementary CTAT Table’
- Do not add subheadings
- Add as many rows as needed to include all information
- Only include one item per row

**If the CTAT form is not relevant to your study, please outline the reasons why:**

|  |
| --- |

- 1. **Antibodies**

| **Name** | **Citation** | **Supplier** | **Cat no.** | **Clone no.** |
| --- | --- | --- | --- | --- |
|  |  |  |  |  |

- 1. **Cell lines**

| **Name** | **Citation** | **Supplier** | **Cat no.** | **Passage no.** | **Authentication test method** |
| --- | --- | --- | --- | --- | --- |
|  |  |  |  |  |  |

- 1. **Organisms**

| **Name** | **Citation** | **Supplier** | **Strain** | **Sex** | **Age** | **Overall n number** |
| --- | --- | --- | --- | --- | --- | --- |
|  |  |  |  |  |  |  |

- 1. **Sequence based reagents**

| **Name** | **Sequence** | **Supplier** |
| --- | --- | --- |
|  |  |  |

- 1. **Biological samples**

| **Description** | **Source** | **Identifier** |
| --- | --- | --- |
|  |  |  |

- 1. **Deposited data**

| **Name of repository** | **Identifier** | **Link** |
| --- | --- | --- |
|  |  |  |

- 1. **Software**

| **Software name** | **Manufacturer** | **Version** |
| --- | --- | --- |
| R | The R Foundation for Statistical Computing | 3.4.3 |
| EZR | Saitama Medical Center, Jichi Medical University | 1.36 |
| Prism | GraphPad Software | 7.0 |

- 1. **Other (*e.g*. drugs, proteins, vectors etc.)**

| **ELISA** | **Supplier** | **Cat no.** |
| --- | --- | --- |
| Human C3a | Hycult Biotech | HK354 |
| Human MASP-2 | Hycult Biotech | HK326 |
| Human complement factor H | Hycult Biotech | HK342 |

- 1. **Please provide the details of the corresponding methods author for the manuscript:**

| **Manabu Hayashi, M.D., Ph.D., Department of Gastroenterology, Fukushima Medical University School of Medicine, 1 Hikarigaoka, Fukushima City, Fukushima, 960-1295, Japan.**  **E-mail: m884884@fmu.ac.jp** |
| --- |

**2.0 Please confirm for randomised controlled trials all versions of the clinical protocol are included in the submission. These will be published online as supplementary information.**

|  |
| --- |
